# Grundlagenwissen zu Phagen und ihrer therapeutischen Anwendung

**DOI:** 10.1007/s00103-025-04051-3

**Published:** 2025-05-02

**Authors:** Christine Rohde

**Affiliations:** Abteilung Bioressourcen für Bioökonomie und Gesundheitsforschung, Leibniz-Institut DSMZ – Deutsche Sammlung von Mikroorganismen und Zellkulturen GmbH, Inhoffenstr. 7B, 38124 Braunschweig, Deutschland

**Keywords:** Koevolution, Lytischer Zyklus, Temperente Phagen, Phagom, Phagenresistenz, Coevolution, Lytic cycle, Temperate phages, Phageome, Phage resistance

## Abstract

Phagen (Bakteriophagen) sind Viren, die spezifisch Bakterien infizieren und zerstören können. Sie sind in der Natur weitverbreitet und spielen eine wichtige Rolle in mikrobiellen Ökosystemen. In der Medizin werden sie als mögliche Alternative oder Ergänzung zu Antibiotika erforscht, sie können zum Beispiel bei Wund‑, Harnwegs- und Lungeninfektionen eingesetzt werden. Anwendung finden dabei einzelne Phagen oder sogenannte Phagencocktails.

Dieser Übersichtsbeitrag zum Grundlagenwissen zu Phagen beleuchtet altbekannte Stichworte aus dem Wissen der Phagenbiologie und moderne Zusammenhänge und Forschungsschwerpunkte und stellt die Wirkmechanismen von Phagen als Grundlage für die therapeutische Anwendung vor. Dabei wird vor allem auf die Phage-Wirt-Interaktion, den Mechanismus der Lyse, die Morphologie von Phagen sowie auf spezifische Methoden zur visuellen Darstellung eingegangen. Als Teil des menschlichen Mikrobioms tragen Phagen in den Schleimhäuten (Mukosa) zur Immunabwehr bei. Auch die therapeutisch nicht einsetzbaren temperenten Phagen werden vorgestellt, die als Prophagen im bakteriellen Genom verweilen können, sowie die in den letzten Jahren entdeckten CrAss-Phagen (Crassvirales) und Lak-Megaphagen. Die bakterielle Phagenabwehr, die Phagenresistenz sowie Phagen-Antibiotika-Synergien sind weitere Themen. Ein Ausblick auf die zukünftige Forschung wird gegeben, dabei wird die Bedeutung der koordinierten Sammlung von Forschungsergebnissen herausgestellt.

Phagen sollen Antibiotika nicht ersetzen, sie können deren Effizienz sogar wieder verbessern. Die Zulassungsprozesse zur Phagentherapie sind aktuell noch herausfordernd. Das Vertrauen in Phagenpräparate muss auf Qualität bauen können, die durch harmonisierte Standards zu garantieren ist.

## Abstract (en)

Phages (bacteriophages) are viruses that specifically infect and kill bacteria. They are very abundant in nature, playing a highly relevant role in microbial ecosystems. In medicine, they are investigated as a potential alternative or supplement to antibiotics and can be used to treat wound, urinary tract and lung infections, for example. Single phages or so-called “phage cocktails” are applied.

This overview article on basic knowledge of phages sheds light on well-known keywords from classical knowledge of phage biology and on state-of-the-art research focuses. Mechanisms of phage activity are presented as a basis for therapeutic application. Particularly, the phage–host interaction, lysis mechanisms, phage morphologies and specific methods for visualisation are discussed. Being part of the human microbiome, phages contribute to immune defence, especially in the mucosa. Temperate phages that are able to reside in bacterial genomes as prophages and therefore not suitable for therapy use as well as the CrAss phages (Crassvirales) and Lak megaphages discovered in recent years are also introduced. Further topics are bacterial phage defence, phage resistance and phage–antibiotic synergies. An outlook on future research is given, emphasising the importance of a coordinated collection of scientific results.

Phages should not replace antibiotics, but they can even improve their efficiency. Currently, the licensing processes for phage therapy are still challenging. However, trust in phage preparations must be based on quality, which has to be guaranteed by harmonised standards.

## Einleitung

Spätestens seit dem Report der Weltgesundheitsorganisation (WHO) zum globalen Fortschreiten der Antibiotikaresistenzkrise 2014 wurde steigendes Interesse an Phagentherapie deutlich, es zeigte sich in den Lebenswissenschaften und der Medizin. Die vergangene Dekade eines regelrechten Momentums der Bakteriophagen als für Bakterien spezifische und diese lysierende Viren zeigte sich in thematisch breit gefächertem weltweiten Interesse der Forschung, v. a. mit dem Ziel der Nutzung des Potenzials der Phagen zur antibiotischen Komplementierung. Beide grundsätzlich verschiedenen Phagentypen, die rein virulent-lytischen und daher therapeutisch interessanten und die temperenten mit 2 möglichen Lebenszyklen, werden insbesondere hinsichtlich der genomischen Ausstattung intensiv beforscht. Letztere sind in der Natur und im Mikrobiom wesentlich häufiger. Beide Phagentypen können doppel- oder einzelsträngige DNA (dsDNA/ssDNA) bzw. RNA tragen, therapeutisch genutzte sind bisher stets dsDNA-Phagen mit einer Kopf-Schwanz-Struktur. Die Phagenbiologie wurde und wird von unterschiedlichster Seite beleuchtet, mit immer tieferem Verständnis und wachsenden Datenmengen, als Prämisse für die Herangehensweisen in translationalen Projekten und zur Ebnung des international und national deutlicher werdenden klinischen Anwendungsweges. Dies spiegelt sich in einer Art neuen Grundlagenwissens über Phagen.

Im vorliegenden Artikel zeigen ausgewählte neue Erkenntnisse kombiniert mit bekanntem Grundwissen auf, wo weitere Forschungsprioritäten gesetzt werden könnten. Der Beitrag erklärt zunächst das spezifische Wirkprinzip der Phagen und ihre lytische Aktivität, gibt einen kurzen historischen Abriss und beschreibt die evolutive Rolle der Phagen in der Natur, ihre Taxonomie und den Umgang mit dem Zugewinn der Datenlage. Die unterschätzte Rolle der Phagen im Mikrobiom und an den Schleimhäuten (Mukosa) wird betrachtet sowie ein Einblick in die Welt neu entdeckter Riesenphagen vermittelt. Dem weitverbreiteten Pathogen *Pseudomonas aeruginosa* und seinen spezifischen Phagen wird ausdrücklich Raum gewidmet. Phagenresistenz als bakterieller Resilienzmechanismus wird oft hinterfragt, darauf geht der Beitrag ebenfalls ein. Zur aktuellen applikativen Forschung gehört das Thema Phagen-Antibiotika-Synergie, das den Beitrag abschließt. Insgesamt soll verdeutlicht werden, wie komplex sich die Phagenbiologie heute darstellt und wo biomedizinische Forschung und Kooperation weiter auf neuem Grundlagenwissen aufbauen können.

## Phagen in der Natur, ihre Evolution und ihr Schlüssel-Schloss-Funktionsprinzip

Die Äußerung von Weitz und Wilhelm [1]: „Wir leben in einem Ozean der Viren“, bezieht sich auf die Viren der Bakterien, die Bakteriophagen (kurz: Phagen). Die Phagenpartikelzahl der Biosphäre wird auf ca. ein 10-Faches der Zahl der Bakterien und auf 10^31^ geschätzt [2]. Jedes Virus benötigt zur Vermehrung eine passende Wirtszelle, hier Bakterien. Phagen kommen vor, wo es Bakterien gibt, sie sind seit Anbeginn bakterieller Existenz treibende Kraft der Evolution. Ohne Phagen gäbe es keine bakterielle Evolution und keine Kontrolle der globalen Bakterienmasse. Prinzip jedes Virus ist die Anpassung an seinen Wirt. In allen Habitaten findet ein antagonistisch-koevolutiver Wettlauf zwischen Bakterien und Phagen statt und damit die Selektion besser angepasster Varianten. Phagen sind fast immer speziesspezifisch, oft sogar für nur wenige Stämme, obwohl es für einige Spezies auch Phagen gibt, die eine größere Zahl an Stämmen erkennen und in natürlichen Habitaten häufiger sind, Beispiele sind *Staphylococcus aureus* oder *Pseudomonas aeruginosa*. Dies ist leider nicht für alle klinisch bedeutsamen Bakterien der Fall, *Klebsiella*-Phagen sind z. B. sehr stammspezifisch, hier zeigt sich ein Limit der Phagentherapie. Bei der klassischen Methodik der Phagentypisierung auf bakterieller Stammebene werden Sets geeigneter Phagen vergleichend zur Wirtserkennung über Lysemuster eingesetzt, bekanntes Beispiel ist die Rückverfolgbarkeit bei Salmonellenausbrüchen [3].

Die Nachhaltigkeit von Phagenpräparaten verdient aus regulatorischen und ökonomischen Gründen Beachtung. Phagen mit nachhaltig breitem Wirtsspektrum sind die attraktivsten Ziele von Phagenproduktentwicklungen in der Human- und Veterinärmedizin, das können Mischungen verschiedener Phagen (Cocktails) sein oder Produkte einzelner Phagen. Die Wirksamkeit über viele Jahre kann dadurch gewährleistet werden, dass ein Phage einen langfristig stabilen (konservativen), „universellen“ Zellrezeptor einer Bakterienart erkennt. Der Aspekt könnte technisch einfach untersucht werden, indem historisch alte Phagen gegen rezente Stämme getestet werden und umgekehrt. Phagencocktails können immer wieder aus unterschiedlichen Phagen zusammengesetzt werden, was bei den oft zitierten georgischen Präparaten der Fall ist. Aus regulatorischer und Herstellersicht wären solche Produktadaptationen aber aufgrund der noch nicht an Phagen adaptierten Regulatorik mit vollumfänglicher Anwendung des bestehenden Arzneimittelgesetzes mit zeit- und kostenintensiver GMP(Gute Herstellungspraxis)-Herstellung keinesfalls zielführend, diese Aussage trifft im Wesentlichen auf Länder mit westlichen Arzneimittelstandards zu.

Phagenspezifität bedeutet Erkennung des Rezeptors zur Adsorption, wobei es eine Rezeptorvielfalt gibt [4]: Bei grampositiven Bakterien sind es oft Zellwandzucker, Polysaccharide, Peptidoglykanreste, Murein, Zellwand-Teichonsäuren, Zellwand-Glykoepitope, Lipoteichonsäuren, Glukose- oder Galaktosekomponenten oder -substituenten. Bei gramnegativen wurde eine größere Vielfalt identifiziert wie Proteine der äußeren Membran (Outer Membrane Proteins, OMP), Lipopolysaccharid-(LPS-)terminale Komponenten, inkl. Zuckerkomponenten, LPS-O-Antigenstrukturen, Antibiotika-Effluxpumpen. Eine Übersicht zur äußeren Membran der gramnegativen Pathogene zeigten Saxena et al. [5].

**Abb. 1:**
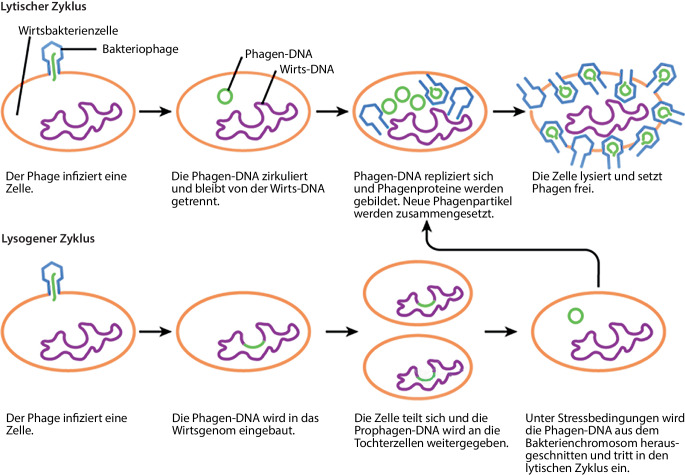
Lytischer und lysogener Zyklus von Phagen. Modifiziert nach [51], Lizenz CC BY-SA 4.0 [52]

Die Schlüssel-Schloss-Funktion der Erkennung von Virus und Rezeptor ist ein Faktor der Koevolution und grundlegend für die zielgerichtete Phagentherapie, bei der der „lytische Zyklus“ passender Phagen genutzt wird (Abb. 1, oben). Er umfasst nach Rezeptorerkennung und Injektion der Phagennukleinsäure in die Zelle einen enzymatisch gesteuerten Ablauf im Bakterium und die nachfolgende Umstellung des bakteriellen Metabolismus zur Vermehrung und Freisetzung neuer Phagen (Propagation). Dabei geschehen Mutationen: Es können besser oder weniger wirksame Phagen entstehen. Phagen können auch an weitere Wirtsspektren angepasst („trainiert“) werden, ein Selektionsprinzip, altbekannt als *Appelmans Protocol, *es wird oft variiert, z. B. auch für den klinisch immer bedeutsameren, häufig Vancomycin-resistenten *Enterococcus faecium* [6].

## Die Geschichte der Phagen

Frederick Twort entdeckte 1915 eine „glasige“ Auflösung von Bakterienkulturen, konnte jedoch die Ursache noch nicht vollständig verstehen. Ihren Namen bekamen die Phagen (altgriech.: „phagein“ = fressen, also „Bakterienfresser“) im Jahr 1917 von ihrem zweiten Entdecker Félix d’Hérelle [7]. Dieser erkannte das Anwendungspotenzial und setzte es klinisch bereits 1919 in Paris um. Phagen konnten wegen ihrer geringen Größe aber erst 1939 durch die Brüder H. und E. Ruska elektronenmikroskopisch sichtbar gemacht werden. So eröffnete sich die Formenvielfalt der Phagen [8, 9]. Ihre Morphologie wurde Grundlage ihrer taxonomischen Einordnung, bis erst kürzlich ein nötiger Paradigmenwechsel stattfand und die binomiale taxonomische Einordnung auf Basis zeitgemäßer Genomik erfolgte (siehe Abschnitt „Morphotypen, Phagentaxonomie, ICTV und PhageDive“). Grundlegend dafür ist eine korrekte Genomanalyse (Phagengenomannotation; [10]).

## Bakteriolyse und Anwendung von Phagenendolysinen

Der bakterielle Zelltod (Bakteriolyse) wird mit der Injektion der Phagennukleinsäure in die Zelle initiiert, gefolgt von der Transkription durch ein Phagen-RNA-Template, Aktivierung spezifischer Restriktionsenzyme zum Abbau der bakteriellen DNA, Zusammenbau von Kapsid und Phagenschwanz mit seinen komplexen Ultrastrukturen, Phagengenomkomplettierung, Kombination von Kapsid und Schwanz, Einschleusung der Phagen-DNA in das Kapsid und die enzymatisch gesteuerte Ausschleusung der neu gebildeten Phagenpartikel durch die Zellmembran nach außen. Im Kapsid eingebettet ist die Phagennukleinsäure.

Die Lyse in Form von Membranläsionen geschieht enzymatisch durch phagenkodierte Peptidoglykan-Hydrolasen. Der Prozess läuft in grampositiven anders ab als in gramnegativen Bakterien, bei denen die innere Zellmembran durch Holin bzw. Pinholin permeabel wird, gefolgt von der Aktivität von Endolysinen und Spaninen, welche die äußere Membran zerstören, Pionierforschung leistete hier Young [11]. Auf die selteneren filamentösen Phagen, die bisher nie in der Phagentherapie Bedeutung hatten, wird hier nicht eingegangen.

Aufgrund der Erkenntnisse zu den lytischen Enzymen entwickelte sich in den letzten Jahrzehnten die Forschung zu innovativen Therapien mit isolierten Phagenendolysinen („Enzybiotics“, siehe auch Artikel von Idelevich und Becker in diesem Themenheft). Phagenendolysine agieren beeindruckend schnell, sichtbar in Time-Lapse-Mikroskopie-Serien [12, 13]. Im Vergleich zur Therapie mit biologisch aktiven Phagen haben Endolysine Vorteile, da ein Phagenproduktionswirtsstamm regulatorisch keine Rolle spielt; bei der Zulassung von Präparationen mit ganzen Phagen werden Produktionswirte, häufig notgedrungen pathogene Stämme, in die Risikobewertung einbezogen. Jedoch ist die Endolysinherstellung bei gramnegativen Bakterien wegen ihrer äußeren Membran biotechnologisch herausfordernd [14].

## Allgemeine Stabilität der Phagen

Beim Zusammenbau der neuen Phagen in einem lysierenden Bakterium wird die spezifische Morphologie, der Bauplan eines Phagen, eingehalten. Ein typischer Kapsid-Morphotyp ist der Ikosaeder (20-Flächen-Körper), der z. B. bei den geschwänzten *Caudoviricetes* vorkommt und eines der stabilsten Bauprinzipen der Natur darstellt, mit einer geringen Oberfläche im Verhältnis zum Volumen. Die physikalische Phagenstabilität ist erstaunlich, meist sind sie auch gegenüber natürlichen pH- oder Temperaturschwankungen sehr tolerant. Sogar höheren Drücken können sie standhalten, was für inhalative Therapien mit Vernebelung von Phagen hochrelevant ist [15]. Aufgrund der allgemein guten Stabilität sind Phagenpräparate ohne erhebliche Titerverluste auch logistisch unproblematisch. Eine allgemein gute physikalische Toleranz und Haltbarkeit hochgereinigter Phagenpräparate konnten nachgewiesen werden und sind in Projekten mit klinischen Studien wichtige Basis. Eine Stabilisierung ist allerdings bei hochgereinigten Phagenlösungen nach Abreicherung aller bakteriellen Zellbestandteile angesagt.

## Morphotypen, Phagentaxonomie, ICTV und *PhageDive*

Auch wenn sich Phagen der 3 häufigsten Morphotypen, die *Caudoviricetes* mit geschwänzten Myoviren, Podoviren und Siphoviren, untereinander sehr ähneln, haben Phagen oft spezifische Merkmale wie elongierte Kapside (Phagenköpfe), Kapsidkragenstrukturen, variierende Schwanz- oder Plaque-Morphologien. Jeder intakte lysefähige Phage verursacht ein Loch im Bakterienrasen, genannt Plaque. Plaque-Morphologien von Phagen sind Erkennungsmerkmale und Plaque-Zahlen nötig für exakte Phagentiterbestimmungen.

Ein Podovirus hat einen sehr kurzen Schwanz, ein Siphovirus einen langen biegsamen Schwanz. Myoviren haben einen kontraktilen Schwanz, bekanntester Vertreter ist Coliphage T4 [16], ein Referenzphage mit vergleichbarer Bedeutung wie *Escherichia coli* K‑12 unter den Bakterien.

2022 löste das „International Committee on Taxonomy of Viruses“ (ICTV) die Ordnung *Caudovirales* auf [17] und bemüht sich um eine neue, den aktuellen Erkenntnissen der Genomik angepasste taxonomische Einordnung von Prokaryotenviren. Ein eindrucksvolles Beispiel ist die wachsende diverse Gruppe der N4-ähnlichen Phagen [18], der Coliphage N4 wurde in den 1960er-Jahren entdeckt, er selbst blieb mit einer großen Gruppe der heute interessanten N4-like-Phagen lange genomisch unbeachtet. Die Forschung zur Diversität der Phagen, zur Interaktion mit Wirtszellen und ihren Struktur-Funktions- sowie Genotyp-Phänotyp-Korrelationen schreitet weltweit in vielen Laboren voran, große Datenmengen, vor allem aus der Genomik, werden erzeugt. Es mangelte bisher an einer harmonisierten, international zugänglichen Datenbank für Prokaryotenviren, welche alle Daten umfassend zusammenträgt. Seit 2022 gibt es die Datenbank *PhageDive* [19], die nun die Daten zu dieser größten bekannten Gruppe der Viren bereitstellt.

## Untersuchung mittels Elektronenmikroskopie

Ein Phagenkapsid ist ca. 30–100 nm groß und im Lichtmikroskop nicht erkennbar. Phagen können wie andere Viren mithilfe der Transmissions- bzw. Rasterelektronenmikroskopie (TEM und REM) visualisiert werden, beide können unterschiedliche Fragestellungen lösen. Mit TEM können die Phagenultrastruktur und die Reinheit eines Präparates im Hinblick auf die Erfüllung von Qualitätskriterien untersucht werden. Ein Phagenpräparat könnte z. B. durch andere Phagen, inklusive potenzieller temperenter Phagen, kontaminiert sein, die nach Induktion aus einem bakteriellen Genom das Präparat kontaminieren und morphologisch auffallen. Temperente Phagen haben das Potenzial zweier unterschiedlicher Lebenszyklen (siehe auch Abschnitt „Das Darmmikrobiom und sein Phagom, temperente Phagen“ und Abb. 1)*. *SEM eignet sich z. B. zur Visualisierung und zur Bestimmung des Zeitpunkts der Adsorption.

Im Vorfeld und während des Projekts *Phage4Cure*^1^, welches die erste deutsche systematische klinische Studie mit Phagen beinhaltet und auf *P. aeruginosa* zielt, wurden Fragen u. a. auch begleitend per EM untersucht, in dem Fall mit *Acinetobacter baumannii* in einem präklinischen *In-vivo-*Maus-Lungenmodell [20]. EM kann diverse Struktur-Funktions-Beziehungen darstellen, auch bei Virus-Wirt-Interaktionen.

## Jumbophagen und spezielle Aspekte der Phagenwirkung

Gelegentlich werden im Zuge von Forschungsprojekten Phagen mit besonderen Eigenschaften entdeckt, z. B. wurde der *E.-coli*-Jumbophage G17 in einem Projekt zu One Health (Triade der Gesundheit von Mensch, Tier und Umwelt) gefunden, er hat ein Genom von ca. 400 kb [21]. Übliche Phagengenome haben zwischen < 10 kb und 175 kb, Jumbophagen jedoch ca. 200 kb bis ca. 500 kb. Der erste Jumbophage, der entdeckt wurde, war der *P. aeruginosa*-Phage „phiKZ“ mit einem Genom von 280 kb [22], er wurde deren Modellphage z. B. zur Erforschung bakterieller Resilienzmechanismen gegen Phagen und zum Verstehen der Wirtsgenexpression unter Phagenmodulation [23, 24]: Transkription, Replikation und Translation laufen teilweise in einer Art Nukleus ab, wobei das Genom durch eine Hülle geschützt wird, teilweise im Zytoplasma, beim Phagenzusammenbau spielt wieder die Hülle des „Nukleus“ eine Rolle (bildlich dargestellt bei Harding et al. [24]). Diese Kompartimentierung könnte auch dem Schutz vor bakterieller CRISPR-Cas-Abwehr dienen.

Jumbophage Paride [25] wurde in vitro als wirksam gegen schlafende/persistierende („deep-dormant persisters“) *P. aeruginosa* beschrieben, der Aspekt schlafender pathogener Bakterien ist im Kontext chronischer Infektionen z. B. der Lunge wichtig und daher auch gerade bei *P. aeruginosa*. Die meisten Antibiotika greifen nur wachsende Bakterien an, hierin liegt ein Problem. 2024 berichtete die Arbeitsgruppe um U. Jenal und S. Hiller des Biozentrums der Universität Basel vom dort entschlüsselten molekularen Mechanismus, der einzelne *P. aeruginosa*-Zellen einer Population in den Schlafstatus versetzt: Eine Mutation aktiviert ein Toxin, das den Energiestoffwechsel dieser Zellen erschöpfen lässt und sie im Schlafzustand für Antibiotika unangreifbar macht. Chronisch Erkrankte mit häufigen Antibiosen sind vermutlich Träger schlafender Persister. Eine Extrapolation des Persister-Problems auf andere Bakterienarten und dessen Verstärkung durch Biofilme werden hier nicht betrachtet.

Harding et al. [24] weisen auf die Besonderheiten der Jumbophagen hin und erwarten interessante Anwendungsaspekte. Die Frage potenziellen Verstärkens (Synergie) oder Inhibierens (Antagonismus) von Phagen in einem Therapiecocktail ist erheblich und beides denkbar, aber bisher wenig erforscht. Phage-Antibiotikum-Synergie (PAS, s. unten) in *P. aeruginosa* wurde für den Jumbophagen Paride und das Antibiotikum Meropenem gefunden [25].

Der phiKZ-ähnliche Therapiephage OMKO1 (kein Jumbophage), ein Myovirus und neuer Modellphage [26], nutzt ein Porin von Antibiotika-Effluxsystemen der äußeren Membran als Rezeptor, was bei den Bakterienzellen evolutiv zu einer Rückbildung der Porine führen kann – ein „evolutiver Ausgleich“ bei der Entstehung multiresistenter *P. aeruginosa* hin zu Antibiotika-sensitiven Varianten, wobei Phagenangriffe und die Entwicklung von Phagenresistenz wieder die Effluxpumpenwirkung verändern können. Die unerfreulichsten Effluxsysteme sind „Mex“ („multi-drug efflux“), da sie diverse Antibiotika aus der Zelle ausschleusen. Hier zeigt sich ein vielleicht universellerer Mechanismus der Reversion zur Antibiotikasensitivität durch Phagenangriffe. Chan et al. [26] postulieren daher, dass antibakterielle Phagennutzung die Erfolgsbeständigkeit mancher Antibiotika verlängern könnte.

## Mukosa und die BAM-Immunität

Phagen sind nicht nur therapeutisch vielversprechend, sondern verkörpern ein grundlegendes Prinzip der Natur. Dass wir sogar in direkter Symbiose mit Phagen leben, verdient Erwähnung: Die Schleimhäute (Mukosa) als Eintrittspforten für Bakterien sind Orte verstärkter Abwehr, dort werden im ganzen Tierreich akkumulierte Phagenmengen gefunden. Die Submukosaepithelien werden durch Phagen gegen Bakterien geschützt, Metagenomanalysen zeigten gar korrespondierende Proteindomänenbindungen von Phagenkapsiden an Mucin-Glykoproteine, wieder ein wichtiges Schlüssel-Schloss-Prinzip, das „BAM-Immunität“ genannt wird („bacterial adherence to mucus“; [27]); die Autoren postulierten eine Koevolution von Mukosaoberflächenbestandteilen mit Phagen im Tierreich.

## Das Darmmikrobiom und sein Phagom, temperente Phagen

Im folgenden Abschnitt bezieht sich der Begriff Mikrobiom aufgrund der vielschichtigen Bedeutung auf das Darmmikrobiom. Das Phagom, der Phagenanteil des Mikrobioms [28], folgt vermutlich ähnlichen Entstehungsmustern wie der bakterielle Populationsteil, die Diversität wird in den ersten Lebenswochen zunächst schmal angelegt [29], hinzukommende kolonisierende Mikrobiota verursachen dann auch eine Induktion von in bakteriellen Genomen ruhenden sog. Prophagen [30]. Auf die Präsenz und die noch nicht vollständig erkannte Rolle temperenter Phagen ist unbedingt hinzuweisen. Temperente Phagen können in ihrem lysogenen Lebenszyklus in das bakterielle Genom integrieren und bis zur Induktion als Prophagen verweilen (Abb. 1) bis zum Eintreten in ihren lytischen Zyklus. Ihre genomische Ausstattung trägt daher mehr genetisches und enzymatisches Potenzial als das der rein lytischen virulenten Phagen. Temperente Phagen sind weit häufiger als rein lytische und sind übliche Bestandteile bakterieller Genome.

Neben den für Anwendungsziele gewünschten obligat lytischen Phagen spielen die temperenten in der Häufigkeit und als Evolutionstreiber in der mikrobiellen Diversität die größere Rolle, auch in unserem Mikrobiom, ein Peptid-„Arbitriumsystem“ zur Kommunikation zwischen Phagen wurde beschrieben, das über den Wechsel zwischen lytischem und lysogenem Zyklus entscheidet ([31]; Abb. 1). Vorstellbar ist ein Aufrechterhalten der Komplexität des gesunden Darmmikrobioms durch die Balance zwischen lytischen und lysogenen Phagen. Auch Arbeitsteilung zwischen Phagen ist beschrieben: *Enterococcus faecalis* V583 z. B. enthält verschiedene Prophagenelemente im Genom, 2 davon können sich zu einem lytischen Phagen kombinieren [32], einer kodiert für Strukturproteine, der andere für Genabschnitte der Infektion, beide zusammen sind für die lytische Infektion nötig.

Die bakterielle Mikrobiomgemeinschaft beeinflusst sicher deren Phagom und umgekehrt. Für das Darmmikrobiom wird eine ausgeprägte Existenz in Biofilmen angenommen, auch dies ist noch wenig erforscht, da Phagenverhalten in vitro meist in planktonischen Systemen studiert wird.

## *CrAss*- und *Lak*-Phagen: neue Phagenfunde im humanen Mikrobiom

Die „CrAss“-Phagen (*Crassvirales*) wurden 2014 über Cross-Assemblierung genomischer Sequenzen humaner Darmmetagenome entdeckt und als abundante (häufig vorkommende) heterogene Phagen in diesen ansonsten unbekannten Sequenzen beschrieben [33]. Auf der Grundlage von Metagenomik und kulturbasierten Methoden wurde *Bacteroides* als Wirt angenommen. CrAss-ähnliche Phagen gehören offenbar fest zum humanen Darmphagom, sie wurden weltweit bei Primaten gefunden [34].

Eine Gruppe sog. Lak-Megaphagen (nach Laksam, Bangladesch) mit Genomgrößen >500 kb wurde in humanen Stuhlproben identifiziert, sie sind vermutlich lytisch und *Prevotella* zuzuordnen [35], diese *Caudoviricetes* haben eine bestimmte genetische Stopp-Codon-Sequenz gemeinsam, ihre Kultivierung ist aber noch nicht beschrieben. Townsend et al. [36] erhoben die wichtige generelle Frage, wie viele große Phagen aufgrund des Größenausschlusses in kulturbasierten Methoden ihrer Entdeckung entgehen. Grenzen der Kultivierungsverfahren zeigen sich in der Mikrobiologie immer wieder.

## Bedeutung der MOI und EOP für die Kinetik und Effizienz lytischer Phagen

Dosisfindung in vitro und in vivo ist bei Phagenanwendung fundamental, ihre Bedeutung zieht sich durch das gesamte experimentelle und spätere klinische Design (prä)klinischer Studien. Die MOI („multiplicity of infection“), das Verhältnis der Anzahl eingesetzter Phagenpartikel zur Anzahl der Bakterienzellen, hat aufgrund der dynamischen multifaktoriellen Kinetik der Phage-Wirt-Entwicklung im Zeitverlauf einen komplexen Hintergrund [37] und ist eine wichtige Größe der minimalen Phagenkonzentration zur Wirtselimination. In vitro werden möglichst geringe MOI angestrebt, auch der Zeitpunkt der Phageninfektion kann sehr entscheidend sein, z. B. bei kapselbildenden Bakterien. Stets ist aber ein Phage-Wirt-System individuell. Eine befallende Bakterienzelle entlässt oft hohe Phagenzahlen, diese Wurfgröße („burst size“) variiert zwischen < 10 und mehreren Hundert. Auch zur Burst Size gibt es komplexe Modellrechnungen [38]. Alle erwähnten Phagen-„Kenngrößen“ sollten immer gründlich untersucht werden, viele In-vitro*-*Daten und Faktoren der Phagenbiologie spielen für Effizienz- und Sicherheitseinschätzungen eine Rolle, selbstverständlich muss jeder Therapiephage eingehend charakterisiert sein. Die EOP („efficiency of plating“) eines Phagen ist die lytische Effizienz im Vergleich zwischen einem definierten Referenzwirt und einem Teststamm-Panel. Ein Phage mit langsamer Lysekinetik kann in einem Phagencocktail wertvoll sein, da er z. B. noch nicht lysierte Bakterienzellen zeitversetzt angreift. Komplexe Fragen vor der therapeutischen Phagenanwendung können nur durch Erfahrung und transparente Wissensvermittlung seitens bereits erfahrener Experten beantwortet werden, da Phagen individuelle biologische Einheiten mit unterschiedlichen Wirkkinetiken sind, dies wird an anderer Stelle dieses Themenheftes behandelt.

## Ein spezieller Blick auf *Pseudomonas aeruginosa*

*P. aeruginosa* bleibt wichtiges Ziel der Infektionsforschung, da der Keim vielfältige Abwehrmechanismen gegen antibakterielle Wirkstoffe entwickelt (siehe auch Abschnitt „Jumbophagen und spezielle Aspekte der Phagenwirkung“), zudem ist er sehr abundant, genomisch extrem anpassungsfähig, kann viele Körperregionen kolonisieren und in Biofilmen persistieren. Pourcel et al. [39] untersuchten die Phagenresistenz von *P. aeruginosa* und verglichen phagensensitive und -resistente Stämme. In den resistenten fanden sie eine auffällige genomische Flexibilität mit Prophagen, die Immunität gegen eindringende lytische Phagen vermitteln. Multiphagenresistente *P. aeruginosa* könnten Selektionsvorteile haben. Die genomische Komplexität stellt vermutlich ein Regulativ bakterieller Resilienz in diversen Habitaten dar, hierzu ist *P. aeruginosa* am intensivsten beforscht. Global angelegte Untersuchungen zeigten zudem Genotyp-Phänotyp-Korrelationen (*Transcriptional Profiling*) im Kontext der Infektion durch *P. aeruginosa* mit seiner ausgeprägten Biofilmbildung [40], auf Biofilme wird hier jedoch nicht näher eingegangen. Es erscheint logisch und bestätigt sich experimentell, dass es für solch abundante Bakterienspezies vergleichsweise besonders viele Phagen in diversen Habitaten gibt, ein Gegensatz zu *P. aeruginosa* wären z. B. andere Bakterien, die bei Lungenbesiedlung große Probleme bereiten wie *Burkholderia cepacia* oder *Bordetella pertussis*. Für die Verbreitungshäufigkeit von Bakterienarten und die ihrer lytischen Phagen als Regulativ in der Natur ist die Erforschung der beteiligten konservativen Phagenrezeptoren interessant.

## Bakterielle Phagenabwehr, Phagenresistenz

Aufgrund der antagonistischen Koevolution von Phagen und ihren Wirten ist die Phagenresistenz von Bakterien ein verbreitetes Phänomen, in vitro messbar. Die Evolution bakterieller Resistenz gegen eindringende Phagen brachte Möglichkeiten für beide hervor. Labrie et al. [41] beschrieben Phagen als die diversesten lebenden Einheiten aufgrund ihrer dynamischen Antwort auf Selektionsdruck, Phagen müssen Gegenstrategien entwickelt haben [42]. Bevor ein Phage zur therapeutischen Anwendung kommen kann und vor der Zusammenstellung eines Phagencocktails ist es wichtig, die Phagenresistenz im Labor entweder per Flüssigkultivierung in Plattenlesegeräten oder auf Festmedien zu quantifizieren und somit vorherzusagen, gemessen wird die sog. BIM-Frequenz („bacteriophage insensitive mutants“), also wie häufig resistente Mutanten in einer Bakterienpopulation im Zeitverlauf (später im Therapieverlauf) vermutlich entstehen.

Zur Resistenzbeurteilung in der Phagentherapie äußerten sich zahlreiche Experten [43]. Millman et al. [44] publizierten eine Übersicht über bakterielle Phagenabwehrsysteme, oft genomische Cluster in Bakterien; einige Abwehrmechanismen zeigen Ähnlichkeit zu humanen Immunproteinen, einige modifizieren Phagenrezeptoren oder die phagenassoziierte DNA-Restriktion, hinzu kommt das CRISPR-Cas-System als bereits gut beforschtes Immunsystem der Bakterien. Hör et al. [45] beschrieben die Aktivierung Ubiquitin-ähnlicher Proteindomänen während der Phageninfektion wie bei der eukaryotischen Virusimmunität, in Alphaproteobakterien stört das Bil-System den Zusammenbau der Phagenpartikel im Bakterium, sodass nichtinfektive Phagenpartikel entstehen. Hör et al. postulierten daher diese Abwehrsysteme als Vorläufer des angeborenen humanen Immunsystems und als universelle antivirale Strategie. Cury et al. [46] beschrieben ebenso, dass bakterielle Phagenabwehrsysteme offenbar über die gesamte Phylogenie konservativ sind. Man kann folgern, dass bakterielle Phagenabwehr im Phagentherapiekontext hochrelevant ist.

## Phagen-Antibiotika-Synergie: PAS

Es ist schon länger empirische Praxis, Antibiotika und Phagen in individuellen Heilversuchen gemeinsam einzusetzen, um synergistische Effekte zu erreichen, bisher in Fällen, in denen Antibiotika alleine versagten. Stellfox et al. [47] beschrieben z. B. einen Patientenfall, bei dem eine kombinierte Phagen- und Antibiotikatherapie im Falle schwerer wiederkehrender Blutstrominfektionen durch Vancomycin-resistente *Enterococcus faecium* eine Reduktion der Bakterienlast im gesamten Gastrointestinaltrakt bewirkte.

Zur PAS laufen viele Untersuchungen in vitro, seltener vermutlich in präklinischen oder klinischen Studien. Mittlerweile wurde oft beobachtet, dass Phagen in ihren Wirten die Antibiotikasuszeptibilität wünschenswert modulieren, sodass PAS aktuell häufig thematisiert wird. PAS ist aber komplex, deutlich mehr Forschung ist nötig im Hinblick auf Antibiotikaklassen, eingesetzte Phagentypen, Bakterienspezies, Dosisschemata beider antibakterieller Strategien und die angewandte Reihenfolge beider im Einzelfall [48]. Wann immer möglich, sollte PAS in präklinischen und klinischen Studien erforscht werden, um unerwünschte Bakterienlast effektiv zu reduzieren und um den Antibiotika wieder zu besserer Wirksamkeit zu verhelfen. Auch hier zeigt sich die Relevanz und Besonderheit der Phage-Wirt-Interaktion.

## Fazit

Heute kann dank ständig neuer Kenntnisse aus der allgemeinen Phagenbiologie und über Genomik aus einer breiten Phagendiversität auch über das Potenzial der Phagen in der synthetischen Biologie [49], über Strategien bakterienfrei hergestellter Phagen [50] und über gentechnisch adaptierte Phagen für effiziente Phagentherapie nachgedacht werden. Zudem werden isolierte lytische Phagenenzyme wichtiger werden. Stets geht es um die Wechselbeziehung Phage–Wirt. Auch wenn das Schritthalten der Zulassungsprozesse mit modernen Herangehensweisen in der Phagentherapie noch herausfordernd ist, lohnt es, neben der klassischen Phagenanreicherung aus natürlichen Habitaten und nachfolgender Produktion die erwähnten neuen Wege zu ebnen. Je größer Biodiversitätssammlungen der Phagen und ihrer assoziierten Wirte sind und die koordinierte Sammlung zugänglicher Daten (s. oben, *PhageDive*), desto Erfolg versprechender wird die therapeutische Anwendung und neue Forschungsansätze in der Phagenbiologie werden motiviert.

## Footnotes

1 Projekthomepage *Phage4Cure*: https://phage4cure.de/de/projekt/.
